# Higher off-target amplicon detection rate in MiSeq v3 compared to v2 reagent kits in the context of 16S-rRNA-sequencing

**DOI:** 10.1038/s41598-022-20573-1

**Published:** 2022-10-01

**Authors:** Mari-Lee Odendaal, James A. Groot, Raiza Hasrat, Mei Ling J. N. Chu, Eelco Franz, Debby Bogaert, Thijs Bosch, Wouter A. A. de Steenhuijsen Piters

**Affiliations:** 1 Centre for Infectious Disease Control, National Institute for Public Health and the Environment, Bilthoven, The Netherlands; 2 Institute for Risk Assessment Sciences (IRAS), Utrecht University, Utrecht, The Netherlands; 3 Department of Paediatric Immunology and Infectious Diseases, Wilhelmina Children’s Hospital/University Medical Center Utrecht, Utrecht, The Netherlands; 4 Centre for Inflammation Research, Queen’s Medical Research Institute, University of Edinburgh, Edinburgh, UK

**Keywords:** Microbial communities, Microbiology techniques

## Abstract

One of the most widely used techniques in microbiota research is 16S-rRNA-sequencing. Several laboratory processes have been shown to impact sequencing results, especially in low biomass samples. Low biomass samples are prone to off-target amplification, where instead of bacterial DNA, host DNA is erroneously amplified. Knowledge on the laboratory processes influencing off-target amplification and detection is however scarce. We here expand on previous findings by demonstrating that off-target amplification is not limited to invasive biopsy samples, but is also an issue in low bacterial biomass respiratory (mucosal) samples, especially when below 0.3 pg/μL. We show that off-target amplification can partly be mitigated by using gel-based library purification methods. Importantly, we report a higher off-target amplicon detection rate when using MiSeq reagent kit v3 compared to v2 (mean 13.3% vs 0.1% off-target reads/sample, respectively), possibly as a result of differences in reagents or sequencing recipes. However, since after bioinformatic removal of off-target reads, MiSeq reagent kit v3 still results in a twofold higher number of reads when compared to v2, v3 is still preferred over v2. Together, these results add to the growing knowledge base on off-target amplification and detection, allowing researchers to anticipate this problem in 16S-rRNA-based microbiome studies involving low biomass samples.

## Introduction

Over the past decade, interest in the role of the microbiota in human health and disease has increased with the development of culture-independent techniques, including 16S-rRNA-sequencing. 16S-rRNA-sequencing is based on targeted PCR amplification of one or more hypervariable regions of the 16S-rRNA-gene, followed by DNA sequencing. This technique has allowed scientists to make inferences on bacterial diversity and community composition, including those inhabiting the respiratory tract^1–4^. At present, no gold standard method in 16S-rRNA-sequencing exists, mainly due to a broad range of applications and a wide variety of laboratory and bioinformatic procedures^5,6^. This highlights the importance of reporting limitations associated with different 16S-rRNA-sequencing protocols.

Two recent studies addressed off-target amplification of host DNA as an underexplored challenge associated with 16S-rRNA-sequencing of low biomass samples^7,8^. Off-target amplification refers to the erroneous amplification of non-target instead of target DNA fragments. This phenomenon occurs at the PCR-amplification step and is typically observed in samples with a high ratio of host-to-bacterial DNA, such as biopsy samples. Off-target amplification is problematic as it theoretically limits the full potential of the sequencing instrument, and obscures bacterial signals with non-bacterial DNA. So far, off-target amplification has been largely overlooked^7–9^, likely because down-stream bioinformatic pipelines typically remove reads that do not meet the length boundaries of the target sequence. In addition, no studies to date have assessed how laboratory processes following PCR amplification impact the detection rate of off-target amplicons.

In a previous study, we assessed how several laboratory techniques impact our ability to characterize the microbiota of low-biomass respiratory tract samples^10^. Among others, we investigated the impact of the sequencing pool purification method (gel-based/AMPure XP) and sequencing kit (v2/v3) used. AMPure XP is a bead-based purification and gel-based purification is an alternative approach where DNA fragments are separated on an agarose gel, followed by excision and purification of desired band sizes. We showed that the concordance in microbiota profiles between MiSeq reagent kits v2 and v3 as well as gel-based and AMPure XP purification methods was high^10^. Based on these findings, we advocate the use of a combination of the AMPure XP purification method and MiSeq reagent kit v3, which was most time-efficient, while generating a higher number of reads when compared to the v2 kit.

In the current study, we build on these findings by focusing on off-target amplification and the impact of four different combinations of library purification methods and MiSeq reagent kits on off-target amplicon detection. We assessed the rate of off-target amplicon detection across a range of bacterial densities for each combination.

## Methods

### Study population

We studied off-target amplification in low-biomass nasopharyngeal samples from healthy volunteers between the age of 0 and 83 years (*n* = 214), which is a subset of samples collected in the context of a large Dutch cross-sectional population-wide study (PIENTER-3)^10,11^. All performed procedures were in agreement with the ethical standards of the institutional and/or national research committee. The study was approved by the Dutch National Ethics Committee (METC Noord-Holland; M015-022). Written informed consent was obtained from all adult participants, and parents or legal guardians of minors included in the study. Nasopharyngeal swabs were collected in 2016–2017 and stored in 1 mL of liquid AMIES medium at − 80 °C.

### DNA extraction and 16S-rRNA-sequencing

**Figure 1:**
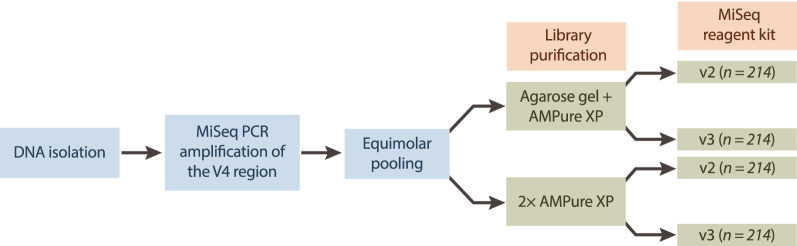
Flowchart of the laboratory procedures performed. Bacterial DNA was isolated, followed by PCR amplification using primers specific to the V4 region of the 16S-rRNA-gene, after which samples were pooled equimolarly. Library pools were then purified (gel-based or AMPure XP) and sequenced using MiSeq reagent kits (v2 or v3). We studied a set of 214 nasopharyngeal samples for each of the four combinations of library pool purification method and MiSeq reagent kits used.

DNA was extracted using the Agowa Mag DNA extraction kit as previously described^10,12^. We amplified the hypervariable region 4 (V4) of the 16S-rRNA-gene using PCR and the 515F/806R-primer pair^10,13^. Besides nasopharyngeal samples, the amplicon pool included DNA extraction (*n* = 14) and PCR controls (*n* = 9). The amplicon pool was either purified using (1) a combination of agarose gel combined with 0.9× AMPure XP magnetic beads (‘gel-based’) or (2) two consecutive purifications using 0.9× AMPure XP magnetic beads (‘AMPure XP’). We sequenced each purified pool using both MiSeq reagent kits v2 and v3 (paired-end; 2 × 250 base pairs [bp] for both kits). As a result, four replicates were generated for each sample, one for each combination (v2/v3 kit and gel-based/AMPure XP purification method; Fig. 1). The concentration PhiX added to each library ranged from 20 to 25%. Samples processed using each of the four combinations were sequenced in separate Illumina MiSeq runs (Illumina Inc., San Diego, CA, US).

### Bacterial DNA quantification

Bacterial DNA was quantified by quantitative PCR (StepOnePlus Real Time PCR System, Thermo Fisher Scientific, The Netherlands) using universal primers targeting the 16S-rRNA gene^10^, which allowed us to assess the relationship between biomass and off-target amplicon detection.

### Bioinformatic processing

For the current study, paired-end reads were processed using DADA2^14^ (v1.16.0; maxEE = 2; truncLen = 200/150) as reported before^3^, allowing inference of amplicon sequence variants (ASVs). Taxonomy was assigned using the naïve Bayesian classifier and the Silva v138 (Version 2; August 2020) reference database^15^.

### Off-target amplification and detection

Although off-target reads are generated during PCR amplification^7^ (‘off-target amplification’), we specifically explored how down-stream laboratory procedures (including library purification method and MiSeq reagent kits) impact the proportion of detected off-target amplicons (‘off-target detection’). Since the target length for the 515F/806R-primer pair is 253 nucleotides (nt), reads outside the normal length range of + 3/− 3 nt (i.e. 250–256 nt) were defined as off-target reads, following the DADA2-tutorial (v1.16)^16^. ASV-sequences were aligned to the human genome (*GRCh38*) using bowtie2^17^ to confirm our read length-based filtering approach.

### Statistical analyses

All analyses were performed in R version 4.1.0 within R studio version 1.4.1717 (Boston, MA). The number and percentage of off-target ASVs/reads was based on data generated through the AMPure XP/v3 kit combination, as previous work indicated this combination was the most time-efficient library purification method, while generating a higher number of reads when compared to the v2 kit^10^. To study the association between read quality and off target detection rate, we assessed the average quality score of the reverse reads (mean over all nucleotides) in relation to off-target detection rate. We used Pearson correlation to study the association between log_10_-transformed bacterial density and read quality, and the log_10_-transformed percentage of off-target reads detected. We also determined the bacterial density threshold at which the bacterial density × off-target detection rate curve starts flattening (*KneepointDetection()-*function from the *GNET2* R-package), indicating the threshold where there is a strong increase in off-target reads. Differences between sequencing runs (v2/v3 and gel-based/AMPure XP library purification) were assessed using mixed linear models, with proportion detected off-target reads as outcome variable, sequence run as fixed effect and subject as random effect. Similarly, we compared differences in read counts between v2/v3 kits before and after bioinformatic removal of off-target reads. *p-*values for pairwise comparisons were extracted using the *emmeans* R-package (v1.6.3)^18^ and were not adjusted for multiple comparisons. The significance threshold was set at 0.05.

## Results and discussion

First, we aimed to quantify the extent of off-target amplification in the nasopharyngeal samples. Using the AMPure XP purification method in combination with the Miseq reagent kit v3^10^, we identified a total of 8978 ASVs, of which 577 (6.4%) were off-target (i.e. read length < 250 or > 256 nt). When incorporating ASV read counts/abundance, these off-target ASVs corresponded with 13.9% of all reads (1.7 M/12.1 M reads). We verified our length-based definition for off-target reads by showing that 99.5% (*n* = 1,661,316) reads aligned with the human genome, indicating a human (e.g. mitochondrial) rather than bacterial origin. Out of these reads, 99.8% (*n* = 1,658,036 reads) were 200 nucleotides long, which is much shorter compared to on-target reads (250–256 nt). In addition, we found a small fraction of reads (0.5% of all off-target reads; *n* = 8428 reads) that could not be mapped to the human genome. Although also off-target (i.e. not within 250–256 read length range), these reads represent a highly dissimilar set of reads, as 99.0% of these reads were longer than 200 nucleotides (range 203–330 nt). We therefore hypothesize that these low-abundant reads represent primer dimers, sequence errors and failed merges of forward and reverse reads.

**Figure 2:**
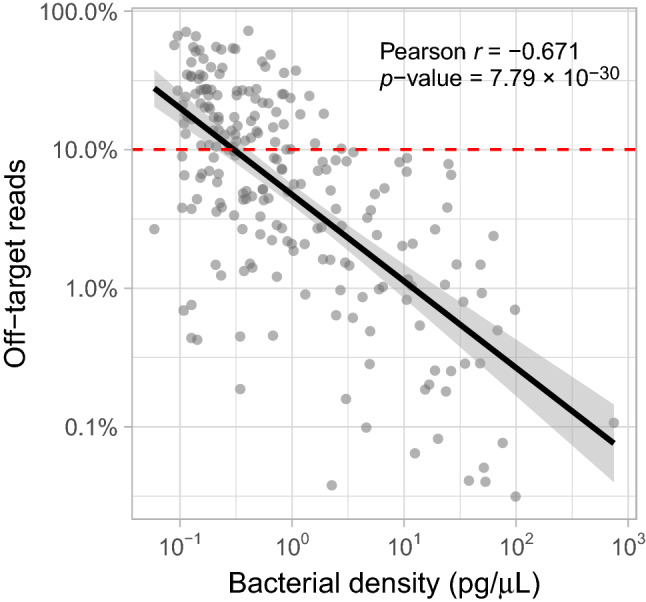
Inverse log–log linear relationship between bacterial density and off-target amplicon detection rate. Shaded area surrounding the black line represent the 95% confidence area. Data were generated using AMPure XP library purification/MiSeq reagent kit v3 (*n* = 214 nasopharyngeal samples). Red horizontal line indicates the threshold at which off-target reads detection is substantially increased.

Previous work by Walker et al. already showed that off-target amplification represents a problem when sequencing 16S-rRNA using V3–V4 primers to determine the microbial composition of invasive human biopsy samples, in which the proportion of human DNA can be over 97%^7^. In our study, we found that off-target amplification also occurs with V4 primers when sequencing non-invasive respiratory mucosal samples (AMPure XP/v3; mean 13.3% off-target reads per sample, range 0.03–72.2%). Adding to previous literature, we were able to show an inverse log–log-linear relationship between bacterial density and the off-target proportion of reads (Pearson *r* − 0.671, *p*-value 7.79 × 10^− 30^; Fig. 2), indicating that the degree of off-target amplification is linked to the bacterial density. Additionally, we find a steep increase in off-target read detection in samples with a bacterial density of less than 0.3 pg/μL, suggesting that off-target read amplification is particularly a problem in samples with a bacterial density below this threshold. A similar relationship was found for the gel-based/v3 combination (Pearson *r* − 0.665, *p*-value 3.67 × 10^− 29^, Supplementary Fig. S1). We speculate that low bacterial biomass may in turn be linked to a high host-to-bacterial DNA ratio, further contributing to the generation of off-target amplicons^7^. This was verified by showing that off-target read amplification is rare in negative (extraction and PCR) controls, suggesting that mitochondrial/human DNA has to be present for off-target read amplification to occur (Supplementary Fig. S2). In addition, we established a negative correlation between the average quality score of the reverse reads (mean over all nucleotides) and the degree of off-target detection (Pearson *r* − 0.392, *p*-value 1.94 × 10^− 9^ Supplementary Fig. S3), likely a consequence of the known association between bacterial density and read quality.

**Figure 3:**
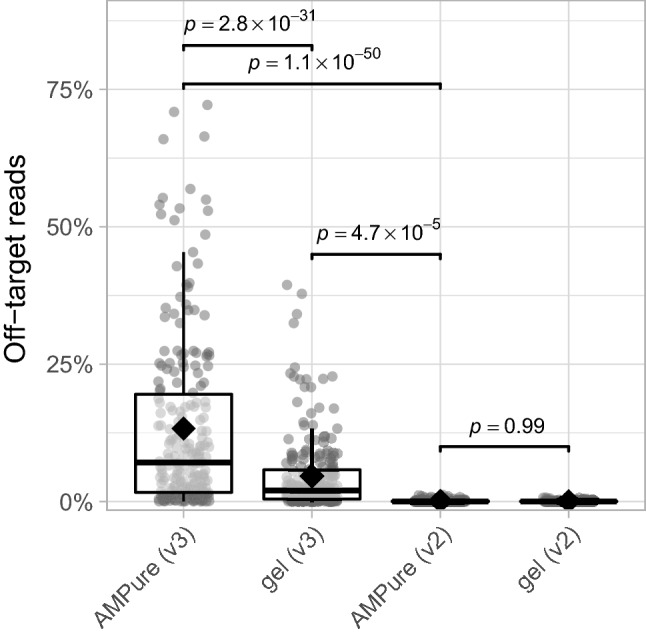
Off-target read percentage across MiSeq reagent kits and library purification methods. Significance was assessed using mixed linear models and the *emmeans*-package^18^ to perform pairwise comparisons. Box plots represent the 25th and 75th percentiles (lower and upper boundaries of boxes, respectively), the median (middle horizontal line), and measurements that fall within 1.5 times the interquartile range (IQR; distance between 25 and 75th percentiles; whiskers). Means are shown as diamonds. *n* = 214 data points for each kit/purification method tested.

Next, we explored the impact of MiSeq reagent kit on off-target amplicon detection. This effect was surprisingly strong, showing an average of 0.1% (range 0.0–1.2%) compared to 13.3% off-target reads per sample for v2 and v3, respectively (AMPure XP-based results; linear mixed effects model; *p*-value 1.1 × 10^− 50^; Fig. 3). The higher detection rate of off-target amplicons by the MiSeq reagent kit v3 was partly mitigated by using a gel-based library purification method instead of only AMPure XP, which resulted in an average of 4.7% detected off-target reads across samples (range 0.0–39.4%; *p*-value 2.8 × 10^− 31^; Fig. 3). This likely results from the more precise extraction and purification of DNA fragments of our target size from other (odd-sized) fragments using gel-based purification versus AMPure XP^19^. Still, we found that the detected off-target reads proportion is much higher for the v3 kit compared to v2 when using the gel-based rather than AMPure XP purification method (*p*-value 4.7 × 10^− 5^; Fig. 3). Since we generated a single PCR amplicon pool of samples in preparation of both v2 and v3 MiSeq sequencing (Fig. 1), we were able to specifically capture the impact of sequencing kit on off-target detection rather than that of the amplification procedure. Although we were not able to pinpoint the exact mechanism behind this observation, we speculate that variation in off-target detection reflects differences in chemicals or sequencing recipe. To our knowledge, this study is the first to report differences in off-target amplicon detection rate between the v2 and v3 kit.

**Figure 4:**
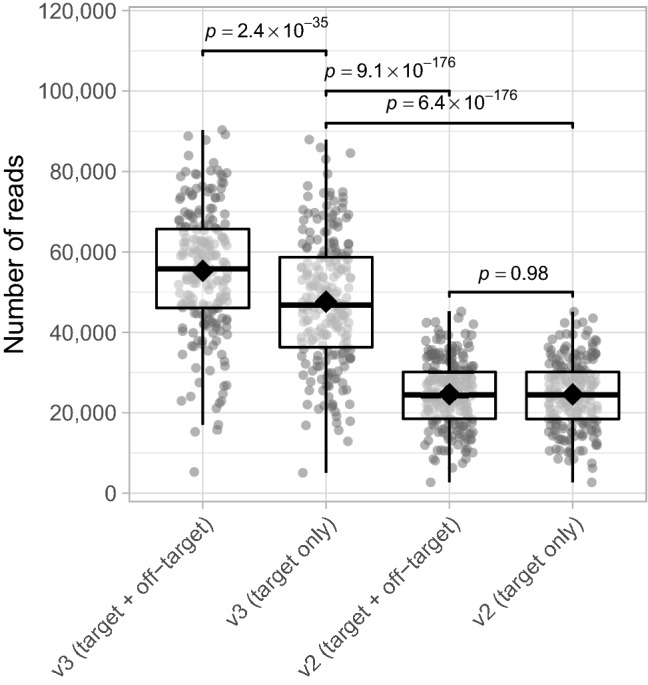
Number of (on-)target/off-target reads for MiSeq reagent kits v2 and v3. Significance was assessed using mixed linear models and the *emmeans*-package^18^ to perform pairwise comparisons. See the caption of Fig. 3 for the definition of box plot elements. Data shown were generated using the AMPure XP purification method. *n* = 214 data points for each group depicted.

According to Illumina, MiSeq reagent kit v3 ensures double the output of a single run (3.3–15 Gb) compared to kit v2 (0.54–8.5 Gb)^20^. We validate this by determining the total number of (on-)target reads for both the v3 and v2 kit using the AMPure XP purification method. Despite the higher off-target reads detection when using MiSeq reagent kit v3 compared to v2, we found that after removal of off-target reads, the number of reads for the v3 kit is still almost twofold higher than v2 (mean fold difference 1.95×, range 1.74–2.22× for matched samples; mixed linear model; *p*-value 6.4 × 10^− 176^; Fig. 4). Similar results were found for the gel-based purification method (Supplementary Fig. S4). Aside from generating a higher number of bacterial amplicons, the MiSeq reagent kit v3 brings forth an improved quality score and increased cluster density, which is why the v3 kit is still preferred over v2^10^.

Together, our findings showed that when characterizing low biomass samples, we currently do not exploit the full potential of the MiSeq sequencer when using the v3 reagent kit as a high percentage of off-target reads is sequenced as well. By implementing a gel-based library purification method we can decrease the proportion of sequenced off-target amplicons, though the problem still persists to a certain degree. We believe our study expands on earlier results of off-target amplification and generated new data on the impact of library purification method/reagent kit on off-target amplicon detection. Although we have established this for the MiSeq sequencer, we are uncertain to what degree this problem occurs with other sequencing instruments. Although shown by us and others for the V4-^21^ and for the V3-V4-primer pairs^7^, we anticipate that off-target read amplification may differ by primer specificity.

Our findings allow researchers to anticipate the loss in reads when planning studies with low biomass samples and/or studies using samples anticipated to have a high host-to-bacterial DNA ratio (e.g. biopsy samples). Researchers can for instance choose to add a lower number of samples to their sequencing run, incorporate gel-based amplicon pool purification or experiment with 16S-primer pairs to maintain sufficient sequencing depth. Caution is especially warranted when working with samples below 0.3 pg/μL density. In addition, we stretch the importance of incorporating a read length-based filtering step in bioinformatic pipelines used in 16S-rRNA-based studies to ensure accurate inference of the microbial composition in downstream analysis. Ultimately, our study may serve as a starting point to further investigate why off-target amplicon detection is more pronounced in the MiSeq reagent kit v3 compared to v2 in low-biomass samples.

## Supplementary Information

Supplementary Figures.

## Data Availability

Raw microbiota data used in this study were made publicly available (PRJNA718293), including minimal participant metadata. Full patient metadata are available upon request. Code used to process and analyze the data is available at https://gitlab.com/wsteenhu/PIENTER_offtarget/. Human genome sequences and annotation files were downloaded from the Illumina iGenomes database (Homo Sapiens *GRCh38* build; NCBI; downloaded 12-1-2021). Taxonomic annotations were based on the Silva v138 (Version 2; August 2020; https://zenodo.org/record/3986799#.YfD5ti-iH0r)^15^.
