# Supplementary material for: Higher off-target amplicon detection rate in MiSeq v3 compared to v2 reagent kits in the context of 16S-rRNA-sequencing
Source: Sci Rep. 2022 Oct 1;12:16489. doi: 10.1038/s41598-022-20573-1 (PMC9526709; doi:10.1038/s41598-022-20573-1)
Supplement: Supplementary file 1 — Supplementary Figures. [file 41598_2022_20573_MOESM1_ESM.pdf]

Higher off-target amplicon detection rate in MiSeq v3 compared to v2 reagent kits in the context of 16S-rRNA-sequencing - SUPPLEMENTARY DATA

Mari-Lee Odendaal[1,2], James A. Groot[1,5], Raiza Hasrat[1,3,5], Mei Ling J. N. Chu[1,3], Eelco Franz[1], Debby Bogaert[1,3,4], Thijs Bosch[1] & Wouter A. A. de Steenhuijsen Piters[1,3]✉

[1]Centre for Infectious Disease Control, National Institute for Public Health and the Environment, Bilthoven, The Netherlands.
[2]Institute for Risk Assessment Sciences (IRAS), Utrecht University, Utrecht, The Netherlands.
[3]Department of Paediatric Immunology and Infectious Diseases, Wilhelmina Children's Hospital/University Medical Center Utrecht, Utrecht, The Netherlands.
[4]Centre for Inflammation Research, Queen's Medical Research Institute, University of Edinburgh, Edinburgh, UK.
[5]These authors contributed equally: James A. Groot and Raiza Hasrat.
✉email: w.a.a.desteenhuijsenpiters@umcutrecht.nl

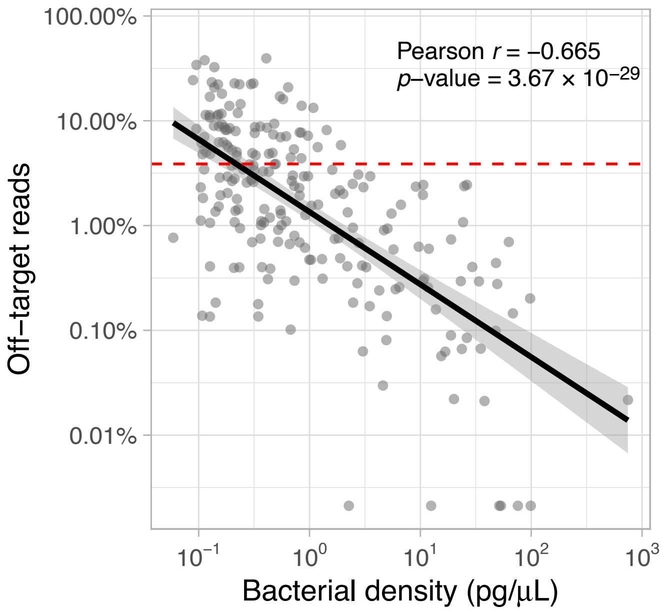

Supplementary Figure S1. Inverse log-log relationship between bacterial density and off-target amplicon detection rate. Shaded area surrounding the black line represent the 95% confidence area. Data were generated using gel-based purification/MiSeq reagent kit v3 (n = 214 nasopharyngeal samples). Red horizontal line indicates the threshold at which off-target reads detection is substantially increased.

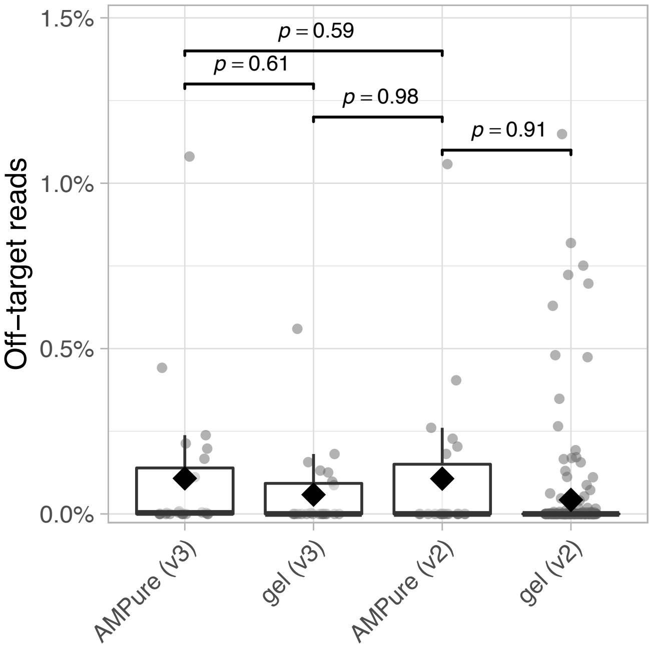

Supplementary Figure S2. Off-target read percentage within the isolation (n = 14) and sequencing blanks (n = 9) across MiSeq reagent kits and library purification methods. Significance was assessed using mixed linear models and the *emmeans*-package [18] to perform pairwise comparisons. See the caption of Figure 3 for the definition of box plot elements. Means are shown as diamonds. n = 23 data points for each kit/purification method tested.

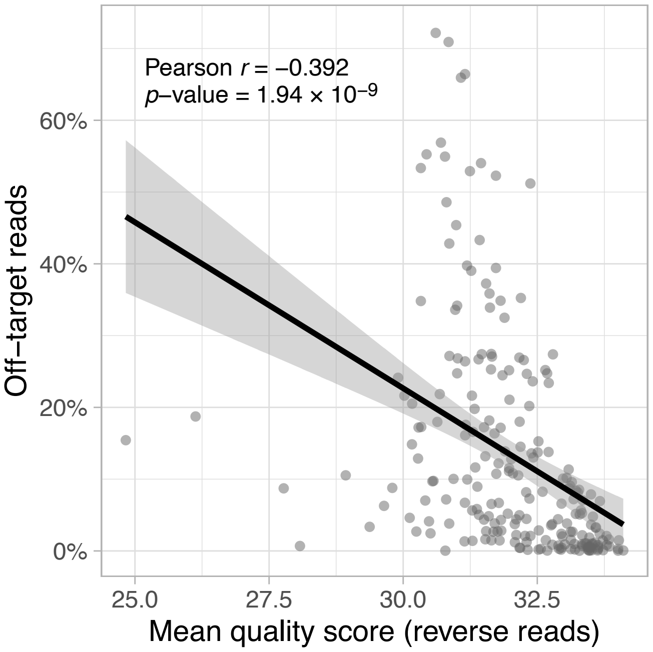

Supplementary Figure S3. Inverse linear relationship between mean quality score of the reverse read and off-target amplicon detection rate. Shaded area surrounding the black line represent the 95% confidence area. Data were generated using AMPure XP library purification/ MiSeq reagent kit v3 (n = 214 nasopharyngeal samples).

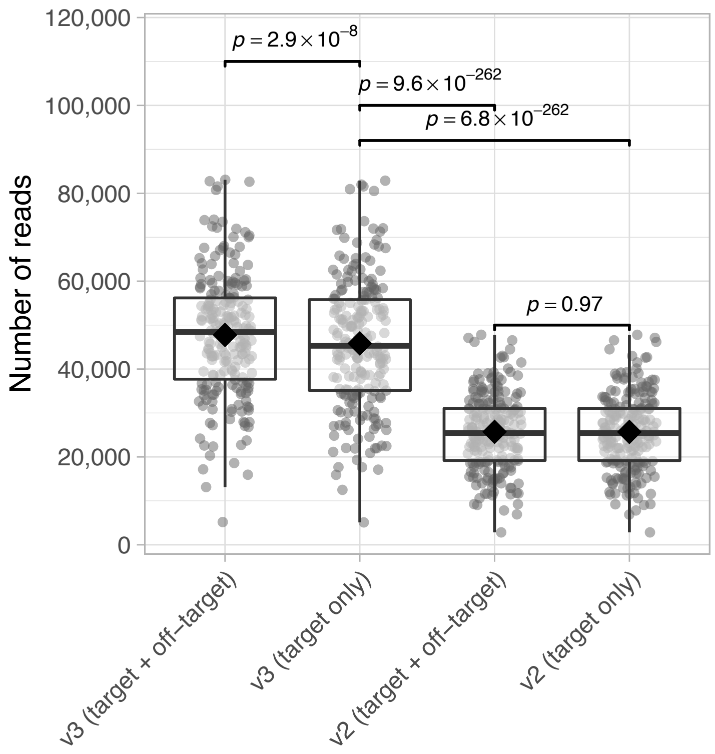

Supplementary Figure S4. Number of (on-)target/off-target reads for MiSeq reagent kits v2 and v3. Significance was assessed using mixed linear models and the *emmeans*-package [18] to perform pairwise comparisons. See the caption of Figure 3 for the definition of box plot elements. Data shown were generated using the gel-based purification method. $n = 214$ data points for each group depicted.
